# Two new, near-infrared, fluorescent probes as potential tools for imaging bone repair

**DOI:** 10.1038/s41598-020-59522-1

**Published:** 2020-02-13

**Authors:** Chien-Chou Lin, Walter Hong-Shong Chang, Tsai-Mu Cheng, Li-Hsuan Chiu, Yen-Hsun Wang, Cheng-An J. Lin, Yuan-Soon Ho, Chun S. Zuo, Yun-Ming Wang, Wen-Fu Thomas Lai

**Affiliations:** 1 Graduate Institute of Medical Sciences, College of Medicine, Taipei Medical University, Taipei, Taiwan; 2 Department of Biomedical Engineering, Chung Yuan Christian University, Chung-Li, Taiwan; 3 Ph.D. Program for Translational Medicine, College of Medicine and Technology, Taipei Medical University, Taipei, Taiwan; 4 Department of Biological Science and Technology, Institute of Molecular Medicine and Bioengineering, Center for Intelligent Drug Systems and Smart Bio-devices (IDS2B), National Chiao Tung University, Hsinchu, Taiwan; 5 McLean Imaging Center, McLean Hospital, Harvard Medical School, Belmont, MA USA; 6 Institute of Graduate Clinical Medicine, Taipei Medical University, Taipei, Taiwan; 7 Department of Research and Department of Dentistry, Taipei Medical University/Shuang-Ho Hospital, New Taipei City, Taiwan

**Keywords:** Optical imaging, Experimental models of disease

## Abstract

A precise imaging technique to evaluate osteogenesis, osteodifferentiation, and osseointegration following peri-implant surgery is in high clinical demand. Herein, we report the generation of two new, near-infrared (NIR) fluorescent probes for use in the molecular imaging of bone repair. The first probe aims to monitor the *in vitro* differentiation of human mesenchymal stem cells (MSCs) into osteoblasts. A NIR fluorochrome was conjugated to a cyclic peptide that binds to integrin α5β1, a factor that promotes osteogenesis in MSCs and therefore functioned as an osteoblast-specific marker. The second probe aims to monitor osteogenesis, and was generated by conjugating the drug pamidronate to a NIR fluorescent gold nanocluster. Pamidronate specifically binds to hydroxyapatite (HA), a mineral present in bone that is produced by osteoblasts, and therefore provides a functional marker for new bone formation. Our results show that both probes bind to their specific targets *in vitro*-differentiated osteoblasts, and not to undifferentiated MSCs, and emit NIR fluorescence for functional detection. This *in vitro* work demonstrates the ability of these probes to bind to active osteoblasts and their mineral deposits and highlight their potential utility as clinical tools for the imaging of the osseointegration process at the molecular level.

## Introduction

Implants that promote bone repair are becoming increasingly important clinically. A key factor that determines the success of such implants is the development of a direct bone-implant interface, referred to as osseointegration. The analyses of transformation of hMSCs into osteoblasts display essential information for osseointegration. Both osteogenesis and osseointegration are typically evaluated by X-ray or by multi-detector computed tomography (CT). However, these imaging techniques are associated with image artefacts that limit their utility^1^. In CT imaging, these artefacts are associated with image reconstruction parameters^2^, X-ray energy and metal composition, and other factors. In X-ray imaging, the occurrence of artefacts depends on the scanner hardware, dense objects, catheters, specimen size, and specimen tissue density^3–5^. Such artefacts prevent clinicians from assessing whether osteogenesis is occurring following bone repair or whether a distortion exists between the implant and surrounding tissue^1,6^. In addition, the scan times required for CT are long, and the resulting radioscintigraphic images are of a low resolution and cannot connect spatial information to cellular activity^7^. As a result, these artefacts and other technical issues can lead to reduce the quality of care. Consequently, a method that can precisely monitor osteogenesis and osseointegration would be of great benefit in clinic

Optical-specific molecular target imaging in the NIR region of the spectrum offers the advantage of high tissue penetration and low tissue auto-fluorescence, resulting in high signal-to-noise ratios during imaging^8^. Optical fluorescence imaging is also being increasingly used to monitor the biological functions of specific targets^9–11^, such as focused ligand targeting in animal models. The developed optical probes which conjugate with a specific ligand led us to design a new method to precisely target new bone formation and osseointegration.

To precisely monitor osseointegration, we utilize the bone differentiation and bone formation factors from the mesenchymal stem cells (MSCs). The optical fluorescent probe is designed to specifically conjugate with osteoblast-differentiated receptor of MSCs, a precursor cell to the osteoblast. The osteoblastic receptor and hydroxyapatite (HA) act as the indicators of osteoblast proliferation and bone formation.

MSCs are multipotent stromal cells that can differentiate into a variety of cell types^12^, including osteoblasts (bone cells)^13^. Human MSCs (hMSCs) can differentiate into osteoblasts under appropriate stimulation conditions^14^. Osteoblasts that become trapped in the bone matrix and remain isolated in the lacunae become osteocytes^15^. Thus, expanding the osteogenic potential and capacity of hMSCs is of a major role for bone regeneration^16,17^. A probe that could indicate the osteoblastic differentiation of MSCs could thus provide an important tool for identifying osteogenic MSCs.

The specific composition of osteoblasts can also be used to target these cells. For example, HA is a specific mineral component of the osteoblast cell surface^18^. Pamidronate (one of several drugs known as bisphosphonates^18^ that is used to treat osteoporosis) can specifically bind to mineral HA and to other minerals, depending on its dosing and molecular structure^19,20^. The ability of fluorochrome-labelled bisphosphonates to bind within the bone matrix is critically important for their use as an imaging tool, which can indicate the degree of new bone formation^21^. A protease-activated NIR fluorochrome coupled with pamidronate has been used to image osteoblasts and osteoclasts using optical tomography^22^.

However, the enzyme cleaved probe relies on a sufficient quantity of disease-induced enzyme. Thus, we designed aggregated types of probes such as the ligand and nanoAu cluster to monitor osseointegration. The optical properties and biocompatibility of gold nanoparticles (nanoAus)^23,24^ also make them an excellent compound to couple to drugs for imaging and therapeutic use. Therefore, the coupling of nanoAu to pamidronate to generate a probe that can target osteoblasts is a promising strategy to enhance efficacy of detecting the osteoblastic differentiation.

In this study, we report the design and creation of two new NIR fluorescent probes: α5β1 L (ligand)-CyTE777 and nanoAu-Pam (Pamidronate). α5β1L-CyTE777 was synthesized by conjugating a new, synthetic cyclic peptide, GG*CRRETAWACGA*, to a modified version of the commercial heptamethine cyanine dye IR-783 (named CyTE777). The cyclic peptide specifically targets the integrin α5β1^25^, which is a highly expressed during osteoblastic differentiation^26^. We hypothesized that this probe can monitor and image osteoblastic differentiation. nanoAu-Pam was generated by conjugating nanoAu particles to the drug pamidronate, and we hypothesized that this probe can be used to monitor osteogenesis. Both probes emitted signals in the NIR spectrum, 674 nm for nanoAu-Pam and 777 nm for α5β1L-CyTE777, and both bound to *in vitro* osteoblasts. These findings demonstrate the specific binding ability of these probes to osteoblasts and highlight their potential utility for the imaging of the osseointegration process at the molecular level.

The developed α5β1L-CyTE777 and nanoAu-Pam probes enable tracking of living osteoblasts and construction of a 3-D image structure, thus providing a non-invasive technique for the detection of the osteogenesis process.

## Results

### Synthesis and characterization of the α_5_β_1_L-targeting probe

The synthetic cyclic peptide GG*CRRETAWAC* specifically binds to integrin α5β1^27^. α5β1 has been previously used as a biomarker to detect the differentiation of MSCs into functional bone-forming osteoblasts^28^.

**Figure 1:**
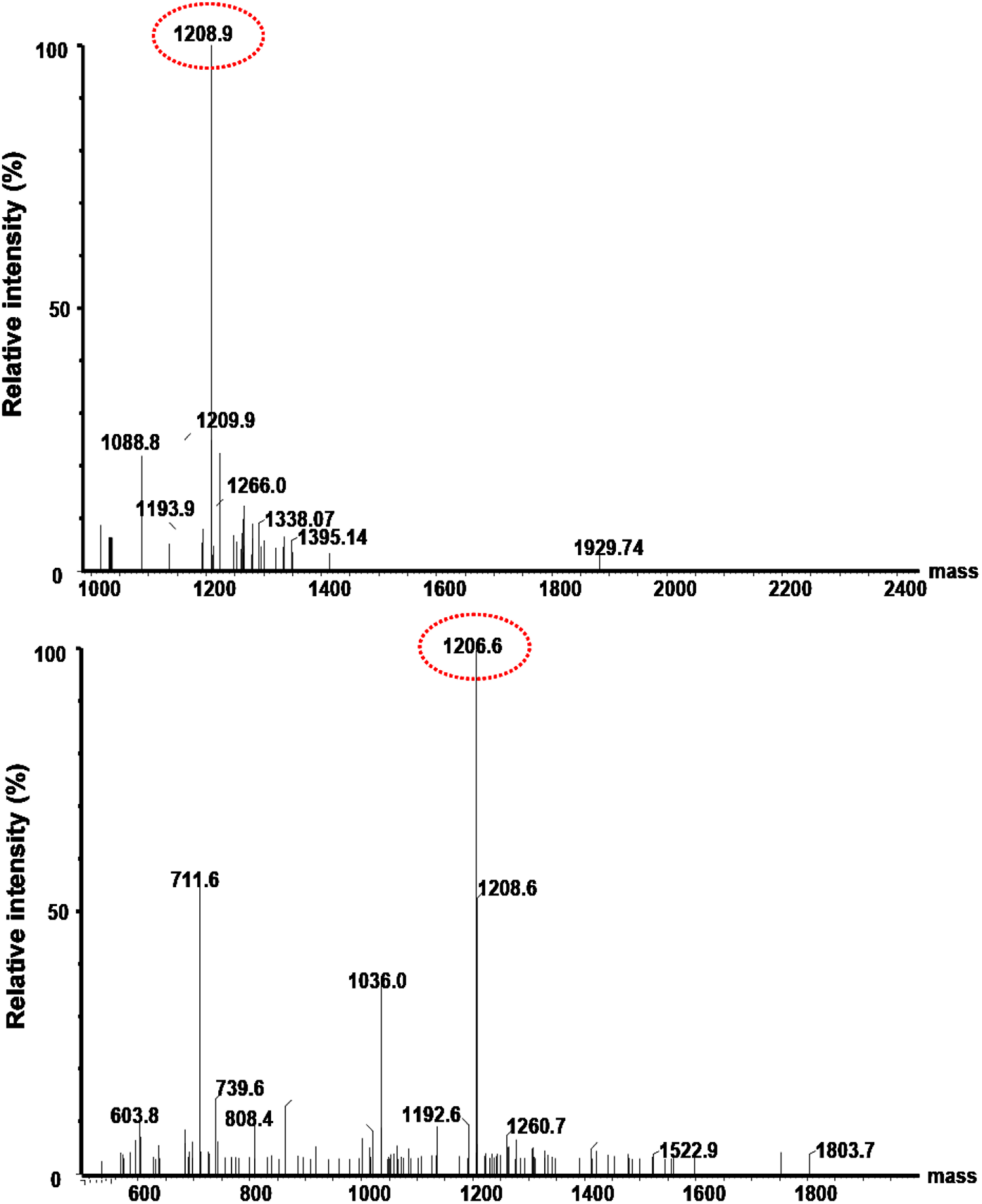
ESI mass spectra of the α5β1-targeting peptides (α5β1 L). (**A**) The linear form of the α5β1-targeting peptide (GGCRRETAWAC) exhibits an intense peak at 1208.9 Da. (**B**) By contrast, the cyclic form of the α5β1-targeting peptide (cGG*CRRETAWAC*) exhibits an intense peak at 1206.6 Da. This result indicates that the linear profile has been modified by dehydrogenization between two cystine residues to create the cyclic form.

**Figure 2:**
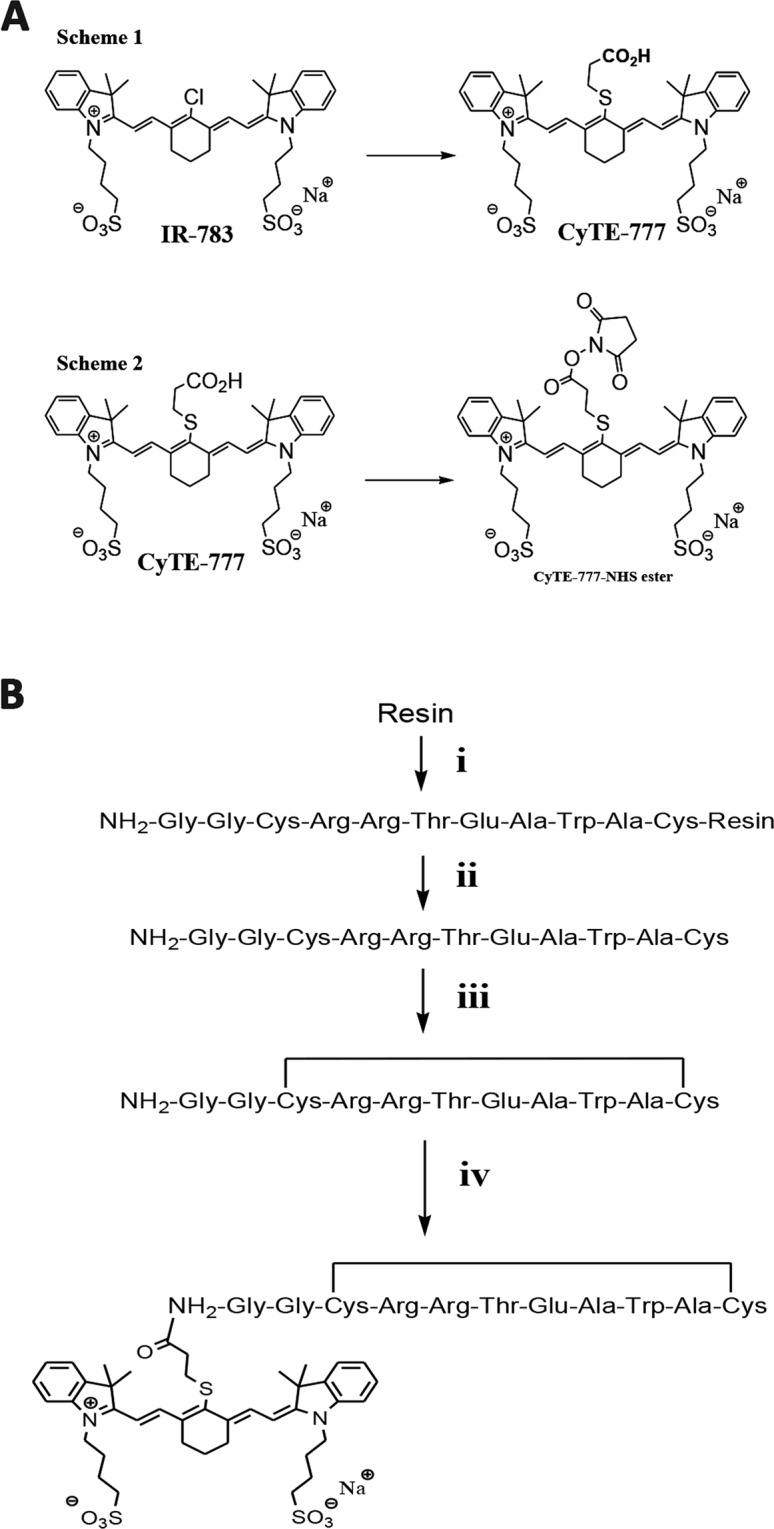
The synthesis of α5β1L-CyTE777. (**A**) Synthesis of CyTE-777 –NHS ester. A carboxyl group was introduced to a solution of the commercially available dye IR-783 to make CyTE-777. (Scheme 1) CyTE-777 carboxylic acid was introduced into a succinimidyl group to form a CyTE-777-succinimidyl ester. (Scheme 2) (**B**) Cyclic µ5ß1 specific peptide conjugation. Conjugation of CyTE777-succinimidyl ester-CONHS to NH_2_ of Gly in cGG*CRRETAWAC* was carried out to form α5β1L-CyTE777.

The linear GGCRETAWAC peptide was synthesized by a solid-phase peptide synthesizer. The synthetic linear GGCRRETAWAC peptide showed a major peak of 1208.9 Da in the ESI mass spectrum (Fig. 1A). Following the formation of an intramolecular disulfide bond between two cystine molecules in the peptide, the major peak shifted to 1206.6 Da (Fig. 1B). This 2.8 Da decrease in molecular weight indicated that the cyclic form of the α5β1-targeting peptide (GG*CRRETAWAC*) was dehydrogenized between the two cystine molecules. The extra glycine molecules generated in the synthesized cyclic peptide (GG*CRRETAWAC*) were used to create a functional N-terminal amide group (Fig. 2A). The final α5β1L-targeting probe (α5β1L-CyTE777) was generated by conjugating the synthetic cyclic peptide (GG*CRRETAWAC*) to the CyTE777NHS ester at this N-terminal amide group (Fig. 2B). α5β1L-CyTE777 exhibited NIR fluorescence at 820 nm with an excitation wavelength of 777 nm. The augmented fluorescence intensity indicated completed conjugation.

### Synthesis and characterization of nanoAu-Pam probe

A bisphosphate-conjugated probe can directly bind to bone surfaces, thus serving as an optimal molecular probe for targeting osteoblasts and osteogenesis^29^ Fe_2_O_3_ nanoparticles conjugated to bisphosphonate molecules have been developed for imaging and therapy^30^, but the bioactivity of nanoAu-tagged bisphosphonate probes is still not fully understood. Recently, nanoAu particles have been used in non-invasive imaging (such as CT)^31^. However, recently developed nanoclustering of gold and iron oxide as a nanoparticles can be prolonged their lifespan and decreasing clearance of reticuloendothelial system (RES) by drug coatings and using biocompatible materials^32^.

**Figure 3:**
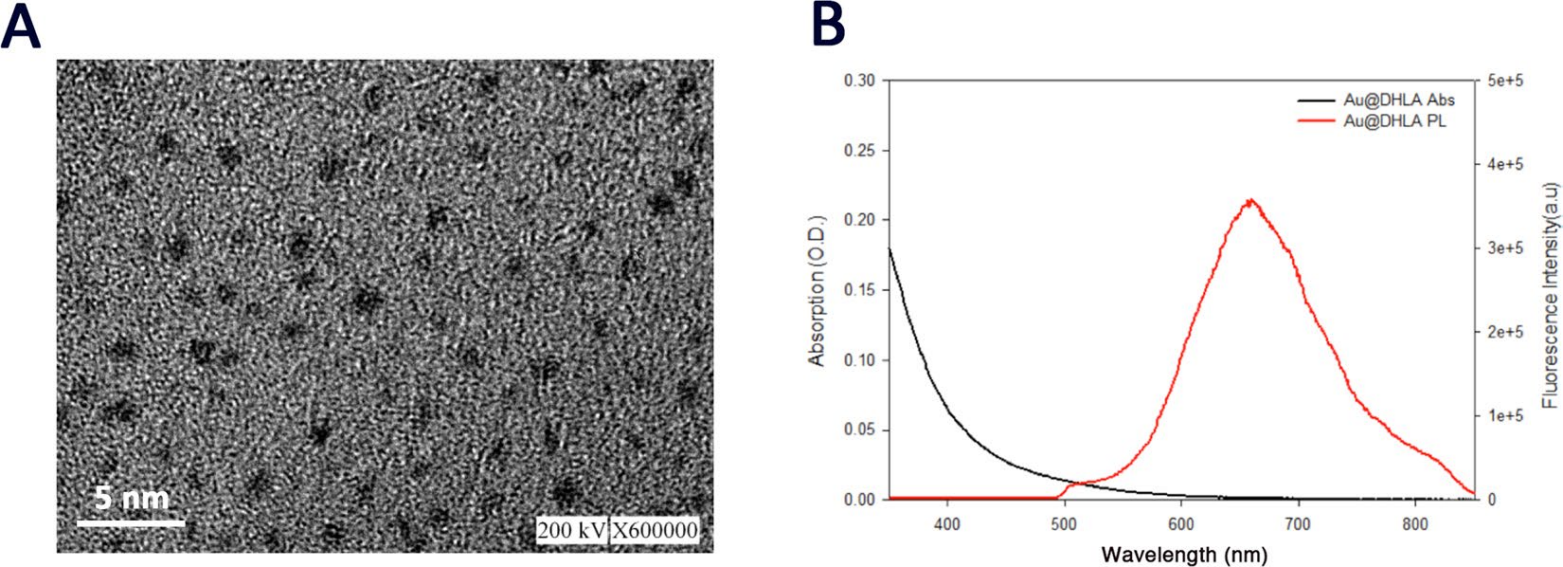
Properties of fluorescent gold nanoclusters. (**A**) A scanning electron microscopy (SEM) image of synthesized gold nanocluster particles (Bar = 5 nm). (**B**) Absorption and photoluminescence spectra for gold nanoparticles. nanoAu itself is a fluorescent molecule, with emission at 674 nm. Abs, Absorption; PL Photoluminescence.

**Figure 4:**
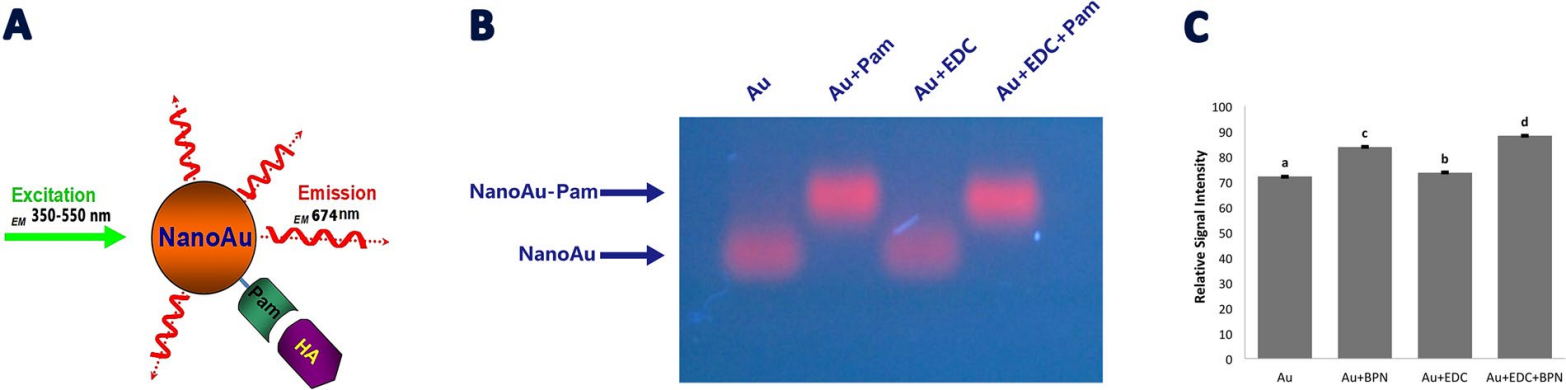
Synthesis of nanoAu-Pam. (**A**) Pamidronate was conjugated onto the carboxylate surface of the nanocluster to generate Pam-conjugated nanoparticles (nanoAu-Pam), which can specifically bind to hydroxyapatite (HA). NanoAu-Pam has emission peak at 674 nm. (**B**) Electrophoretic mobility of nanoAu-Pam in 2% agarose gel. When pamidronate conjugates to the nanoAu, the increased molecular weight of the conjugates decreases nanoAu-Pam mobility, resulting in a band shift on the gel. (**C**) The quantum yield of the conjugated nanoAu-Pam, as measured by fluorescent signal intensity, was approximately 22.3% higher than that of nanoAu alone. Each datum point was from repeated measurements and was presented as mean ± SD. The statistical analysis was performed using One-way ANOVA and Turkey HSD Test.

In this study, nanoAu-Pam targeted to osteoblast-specific HA exhibited a narrow size distribution (1.56 ± 0.3) (Fig. 3A). This narrow size distribution of nanoAu particles contributes a strong NIR emission at 674 nm at a broad excitation wavelength from UV to visible (350–550 nm) (Fig. 3B and Fig. 4A). NanoAu conjugated Pam without or with linker EDC (Au + Pam and Au + EDC + Pam) also showed an increased molecular weight of bands, as demonstrated by agarose gel electrophoresis. Gel electrophoresis also showed that pamidronate-conjugated nanoAu-Pam (Au + Pam and Au + EDC + Pam) had an increased quantum yield, as measured by fluorescence intensity (Fig. 4B,C). These also indicate that the quantum yield of the conjugated nanoAu-Pam was approximately 22.3% higher than that of nanoAu alone. The surface of nanoAu-Pam was coated with DHLA, which has been previously reported to decrease RES clearance, and can increase the probe’s circulation time, accumulation in the bone, and the fluorescence signal intensity^32,33^.

When tested in cell culture, the synthesized nanoAu-Pam bound to osteoblast-differentiated MSCs but not to undifferentiated MSCs.

### α5β1L-CyTE777 binds to osteoblasts *in vitro*

**Figure 5:**
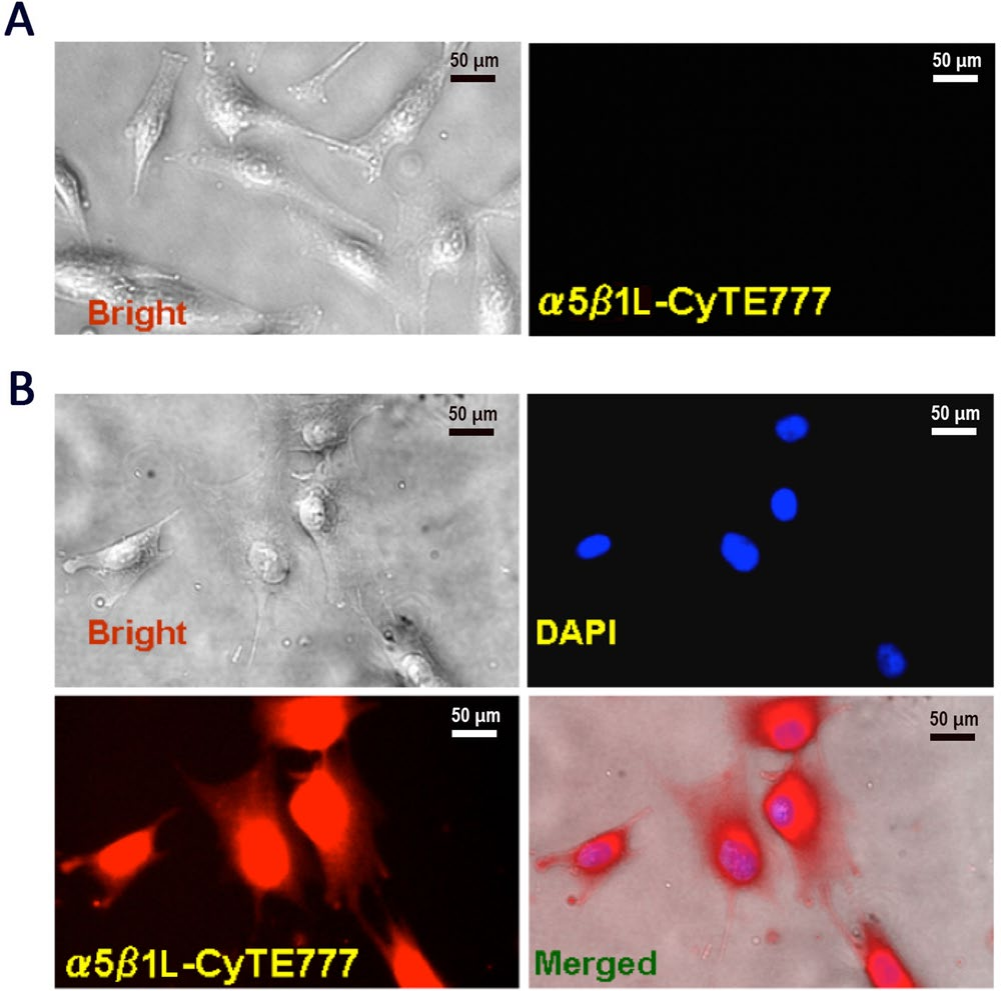
α5β1L-CyTE777 binds to osteoblastic-differentiated MSCs. (**A**) α5β1L-CyTE777 incubated with undifferentiated MSCs in culture does not emit a fluorescent signal. Left, bright-field image; right, confocal micrograph. Bar = 50 µm. (**B**) α5β1L-CyTE777 incubated with MSCs induced to differentiate as osteoblasts emit NIR fluorescence. Upper left, bright field image; right, confocal micrograph (nuclear staining with DAPI is shown in blue). Lower left, confocal micrograph (NIR fluorescence at 820 nm is shown in red), right, merge of bright field, DAPI, and NIR fluorescence images. Bar = 50 µm.

To improve the efficiency of osteoblast targeting, we evaluated the conjugation of an NIR fluorescent dye (IR783) with a synthetic modified α5-binding peptide (cGG*CRRETAWAC*^34^) in the α5β1L-CyTE777 probe to osteoblast cells (Fig. 5). In undifferentiated MSCs, the α5β1L-CyTE777 probe showed minimal signal intensity because of low degree of α5β1 expression (Fig. 5A). However, in MSCs induced to differentiate as osteoblasts with TGF-β1, α5β1L-CyTE777 bound to these cells and emitted a fluorescent signal (Fig. 5B). In agreement with the results of a previous study, which indicated that the osteoblastic differentiation of MSCs can be mediated by α5 (in α5β1) expression^28^, our results demonstrated that α5β1L-CyTE777 specifically monitored osteoblastic differentiation (Fig. 5). In addition, one of the advantages of NIR imaging is the lowest absorption coefficient in the NIR region (650–900 nm) in live tissue targets, such as haemoglobin, water, and lipids^8^.

### NanoAu-Pam binds to HA deposition in osteoblastic-differentiated MSC

**Figure 6:**
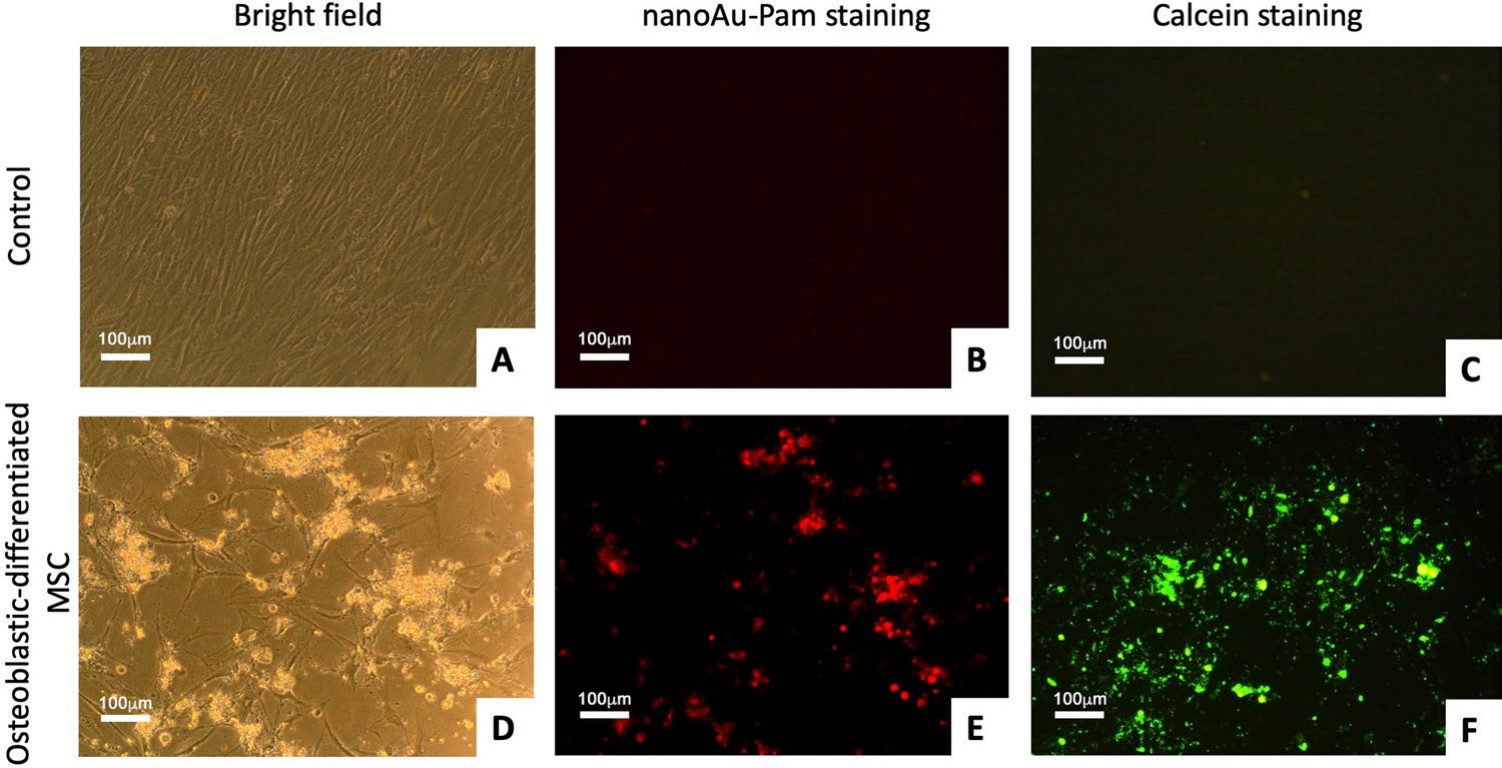
NanoAu-Pam selectively binds to osteoblast-differentiated MSCs.The osteoblastic-differentiated MSC culture showed calcium deposition after 21 days of osteogenic induction (confirmed by positive staining with calcein). These cells were stained separately with nanoAu-Pam and compared to non-induced MSCs (Control). (**A**) Bright field, (**B**) nanoAu-Pam, and (**C**) calcein staining of control cells (non-induced MSCs); (**D**) bright field, (**E**) nanoAu-Pam, and (**F**) calcein staining of osteoblastic-differentiated MSCs. The scale bars indicate 100 µm.

To examine the specificity of nanoAu-Pam in detecting HA deposition/calcification resulting from osteoprogenitor cells bone formation, MSCs were treated with beta-glycerolphosphate in DMEM/LG medium with 10% FBS (osteogenic medium) to induce osteogenic differentiation of the MSCs. After 21 days of induction, nanoAu-Pam probe was applied to target the HA deposition during the osteogenic induction of the culture. It is shown that the osteoblastic-differentiated MSC culture revealed significant level of NIRF signal as compared to the non-induced control cells, indicating that the probe binds to the HA deposition expressed during the osteogenic induction of MSCs (Fig. 6). Calcien stain was also applied to double confirm the HA deposition/calcification of the culture, which also showed a positive stain in osteogenic induction group of the culture. The result indicated that nanoAu-Pam probe specifically targeted the HA-deposition/calcification of osteoblastic-differentiated MSCs and can be detected via NIRF signal.

### NanoAu-Pam binds to osteoblasts *in vitro*

Pamidronate is used to treat bone disorders such as osteoporosis, and specifically binds to the osteoblastic mineral component HA. To assess osteoblastic differentiation, nanoAu-Pam probes were generated by conjugating nanoAu with pamidronate.

**Figure 7:**
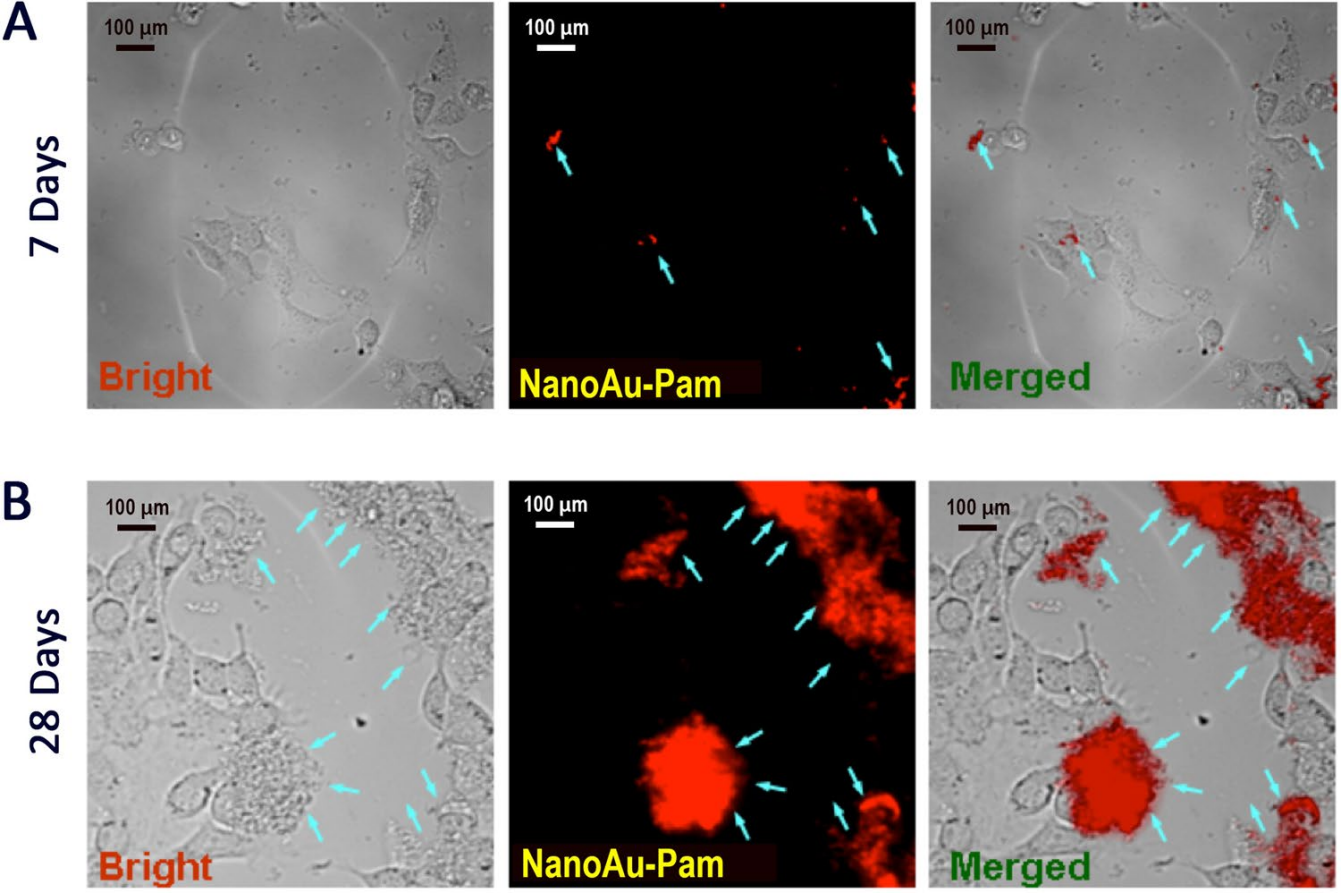
NanoAu-Pam selectively binds to osteoblasts. The specificity and selectivity of nanoAu-Pam were evaluated by incubating the probe with co-cultured human prostate adenocarcinoma cells (LNCaP) and osteoblast cells (7F2). (**A**) A miniature fluorescence of hydroxyapatite (HA) was observed after 7 days in culture. (**B**) After 28 d of culture, nanoAu-Pam exhibited intense fluorescence of HA deposits on 7F2 cells presumed to have undergone osteogenesis. Blue arrows denote the NIR fluorescent probe binds to HA accumulation. The distribution of fluorescence is consistent with the osteoblast cell location. Bars in (**A**,**B**) are 100 µm.

In this study the HA particle was synthesized by a human osteoblast cell line 7F2. In addition, prostate cancer cell lines can express bone morphogenetic proteins^35^. We therefore used co-cultures of the osteoblast cell line 7F2 and the androgen-sensitive human prostate adenocarcinoma cell line LNCaP to test the specificity of the nanoAu-Pam probe. After 28 days of culture, NanoAu-pam was microscopically observed to specifically bind to HA aggregrated-osteoblast 7F2 cells but not to LNCaP cells (Fig. 7). Only minimal particles of nanoAu-Pam accumulated on cell surfaces, as determined by measuring the NIR fluorescence signal, in the osteoblast cells after 7 days of cultivation (Fig. 7A). The 7F2 osteoblasts showed cubic or mild dendritic morphology and had undergone a mild degree of HA aggregation within this time. After 28 days of cultivation, the nanoAu-Pam was bound to HA and found to be deposited on activated osteoblasts (Fig. 7B). Due to the pamidronate binding to osteoblast cell surface proteins at different stages of differentiation^18^, nanoAu-tagged pmaidronate could serve as a probe to visualize, using NIR spectroscopy, osteogenic tissue.

## Discussion

In this study, we conjugated integrin α5β1-targeting peptide with a modified version of the heptamethine cyanine dye IR-783 (Supplement, Scheme 1B). Indocyanine green (ICG) (Supplement, Scheme 1A) is an NIR agent with fluorescent property of absorption at 780 nm and emission at 820 nm. ICG has been approved by the USFDA for clinical applications in angiography and liver function evaluation^36^. Recent studies also reported it to be of minimal cell toxicity in culture^10,37,38^. In this study, we successfully developed a NIR conjugated probe (α5β1-CyTE777), which can significantly monitor the osteogenic differentiation. Considering the ability of NIR signal to penetrate deep tissue and its low auto-fluorescence, this probe design shows potential for clinical detection of new bone formation and osseointegration.

We also report here a second probe, nanoAu-Pam, generated by conjugating nanoAu particles to the drug pamidronate. Because pamidronate specifically binds to the bone mineral HA, this probe has the potential to track human bone repair and osteogenesis^22,29,39,40^. In addition, specific binding to HA indicated the occurrence of osteoblasict differentiation and osteogenesis^41^.

Optical detection of specific molecular or biomarkers *in vivo* has been demonstrated in animal models due to several recent advances, including biocompatible NIR probes, new designs of “smart” enzyme substrate-type probes, and the development of gene-edited photoproteins, etc.^42,43^. These advances have made it possible to understand the functioning of protein networks, to target a precise diagnosis, and to accelerate drug discovery.

Since our novel probes enable effectively to increase the retention, and detectable signal of transplanted cells in osteogenesis, we may also monitor bone healing. Previous research showed that NIR imaging allows for deep signal penetration into tissues in a region of the spectrum where tissue autofluorescence is relatively low, providing high signal-to-noise ratios^8,44^. This nanoAu-Pam probe could have future applications as a “smart” bone repair and monitoring system. Prior studies report osteoblast-imaging probes developed for osteoblastic differentiation and osteogenesis applications in animal models^21,22,45,46^. However, the probes reported in this study are the first two designs that have clinical potential to diagnose different healing stage of the tissue due to their different target molecule, α5β1 and deposited calcium.

These two osteoblast-specific NIR probes offer advantages including a low background noise, high signal penetration, and selective accumulation with a high fluorescence signal from osteoblasts compared with nontarget tissues. Conclusively, the novel NIR probes, α5β1L-CyTE777 and nanoAu-Pam, provide potential platforms for osteogenic differentiation, osteogenesis, and bone repair detection with high sensitivity and specificity. The probe designs may contribute to future diagnostic modalities that can precisely detect the extent of osseointegration of the orthopaedic and dental implants.

## Methods

### Synthesis of α5β1L-CyTE777

#### Synthesis of α5β1-targeting peptides (α5β1L)

A modified integrin α5β1-targeting peptide, GGCRRETAWAC, was chemically synthesized by solid-phase peptide synthesis using the Fastmoc strategy on an ABI 433 A peptide synthesizer (Applied Biosystem, USA). Rink amide resin was washed with N-methylpyrrolidone (NMP) (Sigma-Aldrich, MO, USA) twice, treated twice with a solution of 20% piperidine in NMP for 1 h, and subsequently washed four times at room temperature with dimethylformamide (DMF) (Sigma-Aldrich, MO, USA). In a separate amino acid cartridge, Fmoc-protected amino acid (4 equiv.), *O*-benzotriazole-N,N,N′,N′-tetramethyl-uronium-hexafluoro-phosphate (3.6 equiv.), and *N*-hydroxybenzotriazole (4.0 equiv.) were dissolved in DMF (2 mL), and DIEA (20 equiv.) was added. The active amino acid was added to the resin and allowed to react under nitrogen at room temperature for 1.5 h. The resin was drained and vortexed with acetic anhydride to promote the capping of free amines. After washing with methanol and drying under vacuum, GGCRRETAWAC was cleaved from the resin using trifluoroacetic acid (TFA)/triisopropylsilane (TIS)/H_2_O/1,2-ethane dithiol (EDT) (Sigma-Aldrich, MO, USA) (94.5:1:2.5:2.5, v/v/v/v) for 4 h at the temperature. The cleavage solution was precipitated in 4 °C ether, and the precipitate was dissolved in ddH_2_O to lyophilize.

The intramolecular disulfide bond was formed through air oxidation, followed by vigorous stirring in an aqueous ammonia solution for 3 days at room temperature. After oxidation, the aqueous solution was concentrated and filtered by filter paper to remove the precipitate. The purity of synthetic peptides was confirmed through electrospray ionization (ESI) mass spectrometry.

#### Synthesis of CyTE-777

The heptamethine cyanine dye IR783, which has minimal cell toxicity^37^, was purchased from Sigma-Aldrich (St. Louis, MO, USA). To a solution of IR-783 (250 mg, 0.33 mmol) in 6 mL of anhydrous DMF, 3-mercaptopropionic acid (40.7 μL, 0.467 mmol) and triethylamine (65.3 μL, 0.467 mmol) were added. The green solution was stirred in the dark at room temperature. The reaction was complete after 21 h, as monitored through high-performance liquid chromatography (HPLC). A green solid was isolated through precipitation with 4 °C ether and was washed with cold ether (3 mL). The precipitate was dissolved in water and dried in vacuum ^1^H NMR (300 MHz, CD_3_OD) results were as follows: *δ* 8.89 (d, 2H, *J* = 14.2 Hz), 7.49 (d, 2H, *J* = 7.4 Hz), 7.41 (t, 2H, *J* = 7.6 Hz), 7.34 (d, 2H, *J* = 7.8 Hz), 7.25 (t, 2H, *J* = 7.5 Hz), 6.32 (d, 2H, *J* = 14.3 Hz), 4.19 (t, 4H, *J* = 6.7 Hz), 3.06 (t, 2H, *J* = 7.0 Hz), 2.90–2.87 (m, 4H), 2.70 (t, 4H, *J* = 5.9 Hz), 2.56 (t, 2H, *J* = 6.9 Hz) 2.00–1.92 (m, 10H), and 1.76 (s, 12H). HRMS-ESI [M]^−^ *m*/*z* for C_41_H_51_N_2_O_8_S_3_ 795.2959 was calculated to be 795.5.

#### Synthesis of CyTE777–N-hydroxysuccinimide ester

CyTE777 carboxylic acid (85 mg, 106.65 μmol) (Sigma-Aldrich, MO, USA) was introduced into a Reacti-Vial (Thermo Fisher Scientific, MA, USA) and dissolved in 500 μL of dry DMF. Then, 100 μL of dicyclohexylcarbodiimide solution (Sigma-Aldrich, MO, USA) in dry DMF (220.05 mg, 1.0665 mmol), 186.65 μL of 4-(dimethylamino) pyridine (Sigma-Aldrich, MO, USA) (134.387 mg, 1.1 mmol), and N-hydroxysuccinimide (Sigma-Aldrich, MO, USA) (NHS, 122.74 mg, 1.0665 mmol) were added, and the resulting reaction mixture was protected from light and stirred at room temperature for 12 h. The reaction was checked for completion through HPLC, and the resulting CyTE-777-NHS ester was used without further purification.

#### Synthesis of cGG*CRRETAWAC*-CyTE777

The conjugation of CyTE777-S-ethyl- CONHS to the NH_2_ of glycine in cGG*CRRETAWAC* was performed in a DIEA/DMF (1:9) solution overnight at room temperature. After the solvent was removed, the residue was washed with ether several times at room temperature and precipitated. The product was purified through reversed-phase HPLC.

### Synthesis of nanoAu-Pam

#### NIR fluorescent nanogold cluster preparation

Fluorescent gold nanoclusters were synthesized using nanoparticle etching methods^47^. The particle size of the dihydrolipoic acid (DHLA)-capped nanocluster was analysed using transmission electron microscopy (TEM) (Hitachi H-7100). The DHLA-capsulated nanoAu-Pam was subjected to TEM examination operating at 80 kV using a copper grid.

Pamidronate was attached to the carboxylate surface of a nanocluster using an N-(3-dimethylaminopropyl)-N′-ethylcarbodiimide (EDC) (Sigma-Aldrich, MO, USA) crosslinker. Briefly, nanoAu-Pam (em 674 nm) was synthesized by immobilizing pamidronate (Sigma 2371) onto nanoAu particles using a zero-length crosslinking agent, 1-ethyl-3-[3-dimethylaminopropyl] carbodiimide hydrochloride (EDC). To link pamidronate to nanoAu particles, equal volumes of Au nanoclusters (40 mM), pamidronate (3 mM), and EDC (80 mM) were mixed at room temperature for 2 h for crosslink reaction. To concentrate the conjugated nanoAu-Pam, reaction solution was loaded in a 30-kDa molecular sieve (Amicon) and centrifuged to concentrate the conjugated nanoAu-Pam at 3000 rpm for 10 min. The solution was centrifuged twice and washed with phosphate-buffered saline (PBS) (Sigma-Aldrich, MO, USA) to remove any unbound reactants. UV-visible spectroscopy was used to measure nanoAu-Pam. The purity of synthesis was checked using agarose gel electrophoresis. A UV-2510PC spectrophotometer (Labomed, CA, USA) was used to record UV-visible absorption spectra. The fluorescence intensity of excitation spectra was recorded using an Edinburgh FL 900 CDT Time-Resolved Fluorometer (Edinburgh Analytical Instruments, Livingston, UK).

### Cell culture and specificity evaluation of the probes

A mature osteoblast cell line (7F2), osteosarcoma cell line (MG-63), and prostate cancer cell line (LNCaP) were cultured in α-MEM (Cambrex, NJ, USA) supplemented with 10% FBS (Invitrogen, CA, USA). Cells were cultured without any stimulatory supplements or vitamins in T25 flasks (Nunc, USA) in a humidified incubator at 37 °C using a standard mixture of 95% air and 5% CO_2_. All cells (ATCC; 7F2, ATCC: CRL-12557; MG-63, ATCC: CRL-1427; LNCaP, ATCC: CRL-1740, original from American Type Culture Collection) were obtained from Bioresource Collection and Research Center, Taiwan. MSCs were maintained in 10 ml Dulbeccos Modified Eagles Medium with 1 g/ml glucose (DMEM/LG), 10% FBS, 1 × P/S/F (penicillin/streptomycin/fungizone). The medium was changed every four days. When the cells reached 80% confluence, they were trypsinized and passaged into new dishes at a cell density of 5 × 10^5^ cells/dish.

To evaluate the specificity and binding ability of the nanoAu-Pam in cell models, fluorescent staining of osteoblastic-differentiated mesenchymal stem cells (MSCs) or co-cultured LNCaP/7F2 cells was performed. For osteogenic induction, MSCs were cultured in DMEM/LG medium supplemented with 10% FBS, 100 units/mL penicillin, 100 μg/mL streptomycin, 50 μg/ml L-ascorbate-2-phosphate, 10^−7^ M dexamethasone and 10 mM β-glyceralphosphate in an incubator at 37 °C, 5% CO_2_ culture condition using 24-well plates. After 21-days of induction, the osteoblastic-differentiated MSC culture was stained with nanoAu-Pam or calcein for calcium deposition followed by fluorescent microscopy. Briefly, 10 uM nanoAu-Pam/PBS solution was applied to the cultures for 2 hours in 24-well plate 37 °C, 5% CO_2_ culture condition. After the incubation, cells were rinsed twice with PBS and fixed in 10% buffered formalin. Fixed cultures were evaluated under fluorescence microscopy. Fixed cells were further stained with calcein using standard protocol to confirm the calcium deposition of the positive-stained culture. For calcein staining, 10x calcein/PBS staining solution was added to each well without medium change. The cells were incubated at 37 °C overnight, washed twice by PBS, then subjected to formaldehyde fixation and fluorescence detection.

To examine the specificity of nanoAu-Pam probe in detecting prostate cancer cell-induced HA deposition/calcification, LNCaP (prostate cancer cell line) and 7F2 (osteoblast cell lines) were co-cultured to induce HA deposition/calcification. Cells were cultured in 24-well plate in RPMI 1640 supplemented with 10% fetal bovine serum at 1:1 proportion. After designated co-culture interval, nanoAu-Pam probe was applied to the cultures for the binding of HA deposition. To determine the specificity of α5β1L-CyTE777 probe, MSCs or osteogenesis induced-MSCs were cultured in 24-well plate in DMEM/LG supplemented with 10% fetal bovine serum. α5β1L-CyTE777 probe was applied to the cultures for α5β1 binding. After designated staining time, cultures were wash with PBS twice and subjected to fluorescence detection.

## Supplementary information

Supplement Data.
