# Supplementary material for: Two new, near-infrared, fluorescent probes as potential tools for imaging bone repair
Source: Sci Rep. 2020 Feb 13;10:2580. doi: 10.1038/s41598-020-59522-1 (PMC7018698; doi:10.1038/s41598-020-59522-1)
Supplement: Supplementary file 1 — Supplement Data. [file 41598_2020_59522_MOESM1_ESM.pdf]

Two new, near-infrared, fluorescent probes as potential tools for imaging bone repair

Chien-Chou Lin[†], Walter Hong-Shong Chang[¶], Tsai-Mu Cheng[||], Li-Hsuan Chiu[‡,^],

Yen-Hsun Wang[⊥], Cheng-An J. Lin[¶], Yuan-Soon Ho[†], Chun S. Zuo[‡], Yun-Ming Wang[⊥*],

Wen-Fu Thomas Lai[‡,§,^,*]

[†] Graduate Institute of Medical Sciences, College of Medicine, Taipei Medical University, Taipei, Taiwan;

[¶] Department of Biomedical Engineering, Chung Yuan Christian University, Chung-Li, Taiwan;

[||] Ph.D. Program for Translational Medicine, College of Medicine and Technology, Taipei Medical University, Taipei, Taiwan;

[⊥] Department of Biological Science and Technology, National Chiao Tung University, Hsinchu, Taiwan;

[‡] McLean Imaging Center, McLean Hospital, Harvard Medical School, Belmont, MA, USA;

[§] Institute of Graduate Clinical Medicine, Taipei Medical University, Taipei, Taiwan;

[^] Department of Research and Department of Dentistry, Taipei Medical University / Shuang-Ho Hospital, New Taipei City, Taiwan.

* Corresponding author

Supplementary Information

Supplement Scheme I. Chemical structures of the imaging agents (A) Indocyanine green (ICG) and (B) the Heptamethine cyanine dye IR-783.

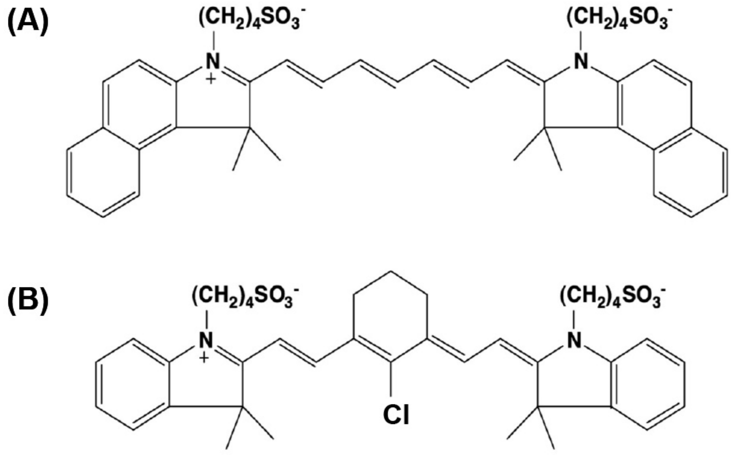

Supplement Figure 1. (A) A viability assay is performed to evaluate the cytotoxicity of the probe on MSCs (Supplement Figure 1). The cells were treated with 2uM or 5uM of nanoAu-Pam in DMEM-LG medium with 10% FBS for 24, 48, and 72hr. After twice of PBS wash, cell viability was evaluated using Trypan Blue staining. The nanoAu-Pam-treated group showed no significant cytotoxicity or inhibition effect on cell proliferation compared to the control group (PBS-treated). Each datum point was derived from triplicate assay and was presented as mean ± SD. The statistical analysis was performed using ANOVA and Turkey HSD Test. (B) Trypan-Blue staining was performed to evaluate the viability of the cells treated with 2uM or 5uM of nanoAu-Pam in DMEM-LG medium with 10% FBS for 24, 48, and 72hr. The nanoAu-Pam-treated group showed no significant amount of trypan-blue positive-stained cells were observed as compared to the control group (PBS-treated).

(A)

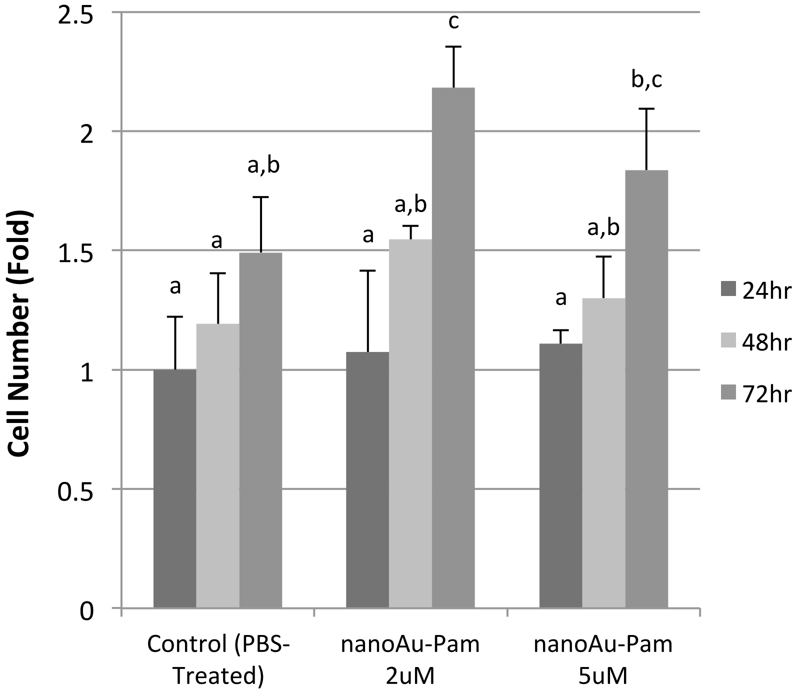

(B)

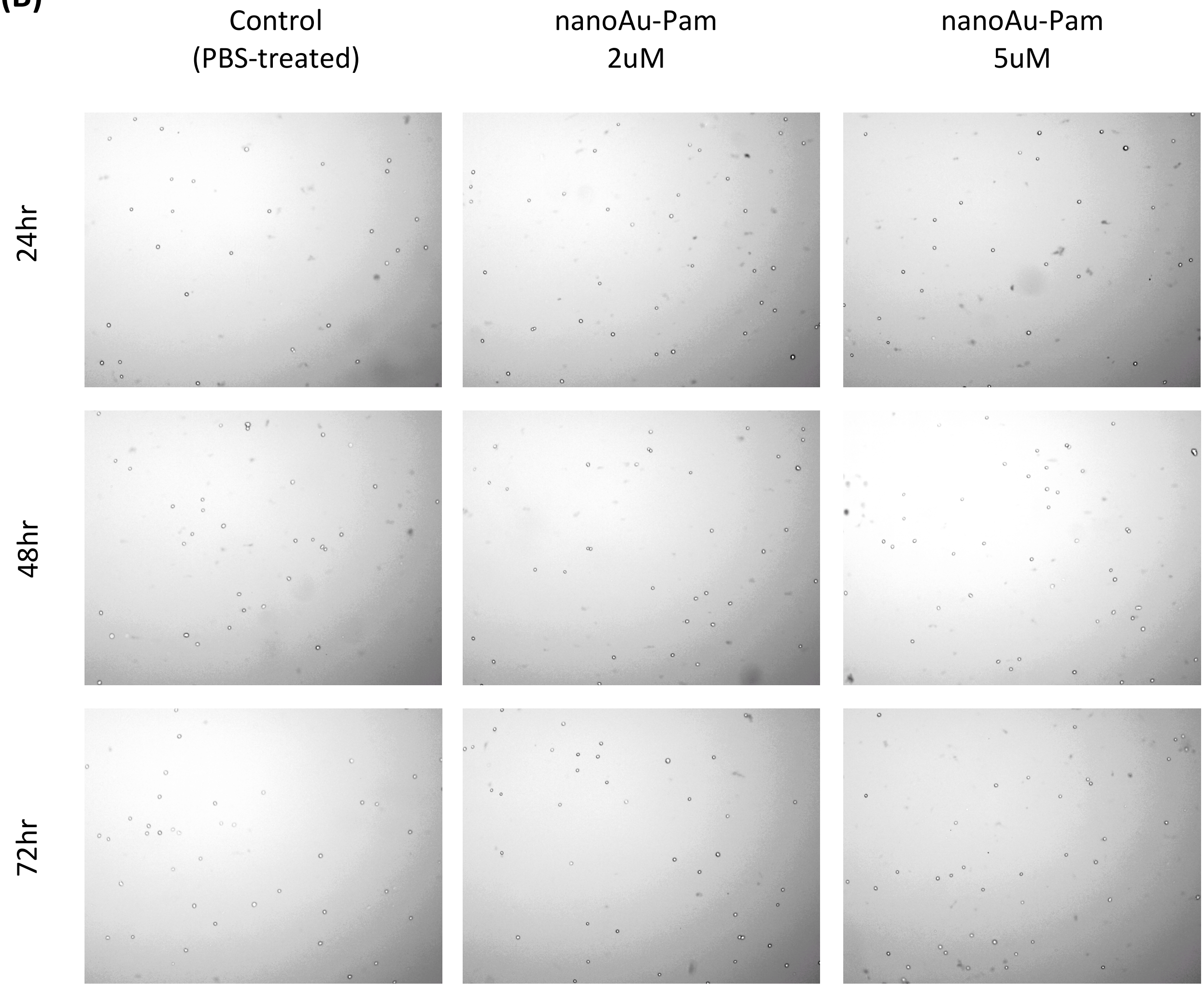
Control
(PBS-treated)
nanoAu-Pam
2uM
nanoAu-Pam
5uM
24hr
48hr
72hr
